# Reaction to fire, thermal, and mechanical properties of materials based on recycled paper granules bound with starch and clay mortar

**DOI:** 10.1016/j.heliyon.2024.e24510

**Published:** 2024-01-17

**Authors:** Lydie Marcelle Thieblesson, Răzvan Calotă, Nastasia Saca, Adrian Simion, Ilinca Năstase, Alina Girip

**Affiliations:** a U F R Environnement/Laboratoire des Sciences et Technologies de l’Environnement Ivory Coast, Cote d’Ivoire; b Technical University of Civil Engineering Bucharest Romania, Romania; c INCERC Institute Bucharest Romania, Romania

**Keywords:** Recycled paper, Starch, Clay, Reaction to fire, Thermal conductivity

## Abstract

The objective of this work is to produce an ecologically friendly material for use in Ivory Coast's construction sector in the future. These materials should have good thermal qualities and be flame resistant in addition to helping to achieve interior comfort. The fundamental components under consideration are freely accessible in Ivory Coast and include clay mortar as a fire retardant, potato starch as a binder, and recycled paper granules as a filler. The suggested ecologically friendly material's manufacturing process is fully described in detail. After conditioning, the team created multiple samples, taking into account that each test that the materials are put through requires various probe sizes for the thermal conductivity test, the reaction to fire test, and the flexural strength test. The best result regarding thermal conductivity of composites was obtained when 10 % clay is added in the mixture, namely between 0.057 … 0.068 W/(mK). During the ignitability tests the flame did not propagate to a height greater than 15 cm throughout the 60 s test time, so it can be concluded that the materials match minimally in the class E of reaction to fire. The flexural strength of tested materials was under 0.8 MPa.

## 1 Introduction

According to the United Nations, the building sector is extremely energy-intensive, accounting for 36 % of world final energy consumption and 39 % of energy-related carbon dioxide (CO_2_) emissions, accounting for about one-third of global primary energy [[1], [2], [3]].

This enormous energy is expended from raw material development to implementation, use, and elimination, as well as the time required to decay at the end of life and curently is taken into account by the cradle-to-grave approach, a model in the scientific method Buildings’ Life Cycle Assessments (LCA) [4].

The life cycle of building materials greatly influences the environment, construction sector by nature is not an environmentally friendly activity [5].

In addition to this problem, there is also the question of household waste management. Starting with the Rio 1992 Summit, environmental protection has been considered a collective concern. To solve this problem, the promotion of environmental management and the mission of sustainable development have put a high demand on all industries, including construction, to adopt proper environmental protection techniques.

Sustainable waste management necessitates the creation of added value through the generation of wealth through recovery and recycling operations. As a result, garbage should no longer be regarded as "something to be discarded," but rather as a resource to be utilized. Sustainable waste recovery means to convert it back into building materials [6]. Reduction, reuse, and recycling are the three fundamental processes by which the economy is created [7]. According to Ghisellini et al. [8], waste management has grown to be a significant component of the circular economy as a means of resource recovery and impact prevention on the environment.

The examples of recycled materials being used in construction were provided while taking the circular economy's guiding principles into account were given by Rybak et al. [9].

The policy governing the use of these materials is increasingly based on national and international legal norms. The study brings attention to the fact that recycled materials are being used in modern buildings more and more properly, which could assist to increase the participation of the circular economy in the process of implementing this topic.

Due to its good mechanical and thermal properties, and within the context presented above, paper brick is a suitable building material to be considered for future projects [10]. The developers created a construction material with adequate mechanical qualities and good thermal insulation properties by using recycled paper as the binder and aggregate.

In polyvinyl chloride (PVC) panels, recycled wastepaper (RWP) was combined with phase change material (PCM) in weight ratios of 25 %, 50 %, and 75 % by Abdulmunem et al. [11]. This material was tested experimentally as an inner wall covering for buildings, and the results indicated that these combinations of new insulation materials in buildings offered good acoustic and thermal insulation and decreased the cooling and heating load of the buildings.

An experimental test [12] was performed on seventeen mixture sets consisting of light weight bricks built with the presence of various additive materials such as expanded perlite (EP) and recycled paper sludge (RPS). The compressive strength values of all samples are around 10–27 MPa. Also, the samples with high additive content (especially 10%RPS+5%EP sample) exhibited the lowest thermal conductivity value (0.432 W/mK) due to the low firing temperature.

Brick samples were examined for their physical traits including bulk density, apparent porosity, and water absorption as well as compressive strength, thermal conductivity, and microstructural features.

However, due to the increasing frequency of fires in various residential and industrial structures around the world, which have disastrous impacts on persons and other resources, caution must be maintained [13].

Thus, all new building materials made from the reuse of materials obtained from demolitions or other types of wastes should be tested for reaction to fire so that the fire risk to be diminished [14,15]. These waste materials will save natural resources due to the high percentage of older buildings. In the near future material from demolitions will be a major problem due to the need for storage. The young civil engineering society must be educated in the direction of the future to use the eco-friendliness materials considering this as the most important factor. This will save natural resources that would have to be used to produce new building materials.

The goal of this work is to create new environmentally friendly material for future usage in the construction industry in Ivory Coast. These materials must be flaming retardant and also should contribute to achieving interior comfort by possessing good thermal properties. The basic materials under consideration are easily available Ivory Coast and consists of recycled paper granules as a filler, potato starch as a binder, and clay mortar as a fire retardant. Potato starch is a readily available natural resource, and clay mortar was chosen for its flame resistance properties [16]. In Ivory Coast, clay is a mineral resource so far exploited mainly for artisanal pottery, yet these resources can be transformed into basic building materials for walls, roofs and floors [17] to provide a solution to the interior comfort of habitats.

Portland cement, the most extensively used and well-known binder, is still the focus of various scientific and technological studies in order to improve its usage qualities, durability, and production cost. Furthermore, its production consumes a high amount of energy and emits a lot of greenhouse gases (CO_2_), which is a major worry when it comes to meeting environmental standards [18]. Clay and starch are environmentally friendly binders that can be used for certain composites as a substitute for Portland cement, which has a problem when it comes to complying with environmental standards.

An alternative based on waste materials in the manufacturing of new and modern products for buildings is not only beneficial to the environment in terms of size waste paper discarded, but it is also a way to secure significant cost savings.

In addition, in various research investigations, the partial substitution of a certain amount of Portland cement by minerals available at a cheaper cost has shown advantageous, not only economically and environmentally, but also in terms of performance. Although clay resources can be found in abundance across wide portions of West African countries, their utilization is still quite limited [19]. Portland cement or clay with recycle paper fibre can be used in construction materials to reduce density, an alternative building material for homes [20].

In this materials all of the steps required in the manufacturing of the proposed environmentally friendly material are detailed, beginning with the actual fabrication phase through mixing the components, conditioning and ending with the actual testing of its performance. The utilization of materials based on recycled paper granules bound with starch and clay mortar represents a groundbreaking and eco-conscious approach that not only minimizes waste but also enhances sustainability in construction and design and it represents a direction that has been very little investigated so far in the specialized literature.

## 2 Materials and methodology

### 2.1 Materials

**Fig. 1:**
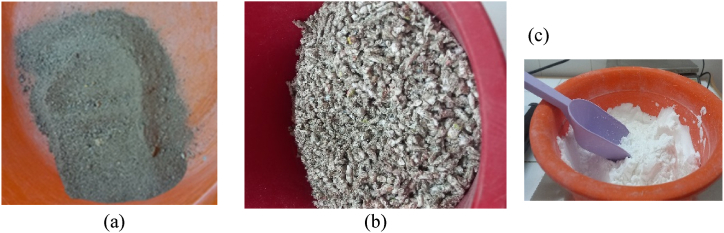
Raw materials a) clay mortar b) paper granules c) potatoes' starch.

This section briefly describes all the necessary steps in order to obtain the innovative material. The raw materials presented in Fig. 1- consist of a filler (recycled paper granules) made from wastepaper (b), the binder is composed of potato starch and the clay mortar is added for mechanical and protection to fire reactions (a,c).

**Fig. 2:**
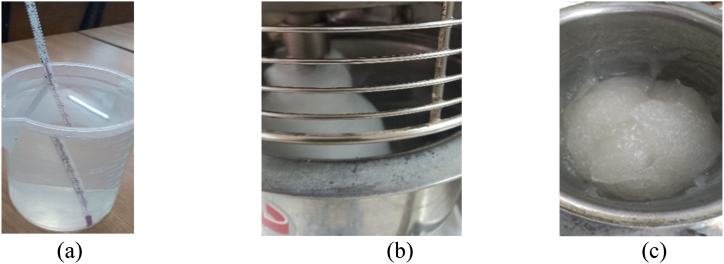
Preparation of starch paste (a) water at the required temperature; (b) starch mixing; (c) starch paste.

The first step of the process is to make a starch paste. The following procedures are depicted in Fig. 2. The measured amount of starch is added to the mixing bowl. The determined quantity of water is heated to 70 °C (a) and gradually added to the starch powder while continually mixing (b) to obtain first the starch milk and then a transparent starch paste known as starch paste (c).

**Fig. 3:**
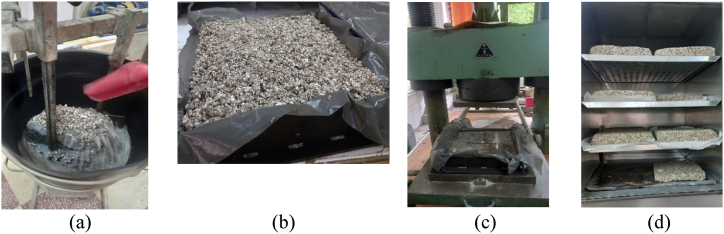
Preparation of the final product (a) mixing of paper granules and mortar of clay and starch paste; (b) molding (paper granule + mortar of clay + starch paste); (c) compression at 10 kN; (d) drying of composite in the oven at 60 °C.

The second step is dry mixing of the clay mortar and the paper granules. Fig. 3 displays the details. The two products resulted from the first and second step are mixed (a), and the final product (paper, clay mortar and starch paste) is put in a mold of size 30 × 30 × 6 cm (b), compressed to 10 kN for uniformization of surfaces and homogenization (c) unmolded and put in the oven at 60 °C for drying and conditioning (d).

The different quantities used for the composites’ elaboration are essentially based on a feasibility study carried out in advance, concerning the elaboration of composites containing only recycled paper granules and starch [6]. During the aforementioned study, the ideal water/starch was 3.3 and recycled paper/starch ratio was 4.17 respectively. These values gave the best results in view of the physical, mechanical and hygrothermal characteristics and were considered as baseline for the present work. By keeping the fixed ratios, different amounts of clay were added. Thus, the clay contribution was taken 10 %, 25 % and 50 %. Also, a 0 % was taken into account as reference.

Following the fabrication of the materials to be examined, the next step was conditioning them to achieve a constant level of humidity within the samples. In order to have a clear picture of how the materials behave during the various tests to which they are subjected from each composition 0 %, 10 %, 25 % and 50 % three identical samples were produced.

**Fig. 4:**
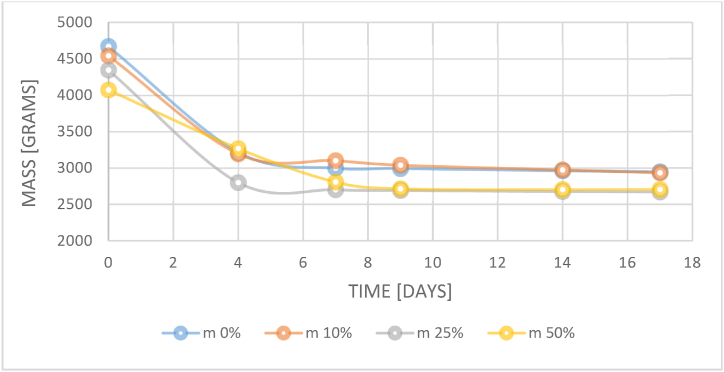
Samples' conditioning period.

After an averaged period of 18 days the amount of water in the material stabilized as shown in Fig. 4 and thus the material was considered to be conditioned and ready for reaction to fire, thermal, and mechanical properties evaluation.

Dimensional measurements revealed no material shrinkage between the start and completion of the conditioning period.

**Fig. 5:**
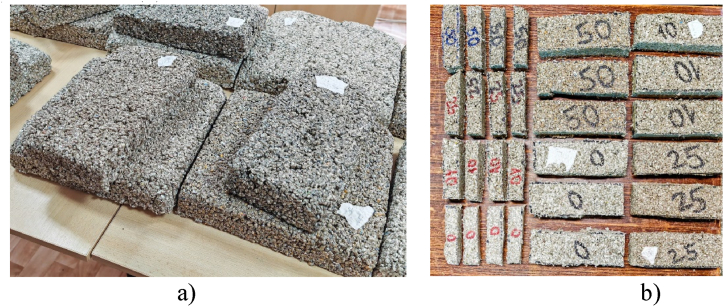
(a) 30 × 30 × 6 cm; (b) 25 × 9x6cm and 16 × 4 × 6 samples.

Fig. 5 illustrates the various geometries necessary for the following testing method. Taking into account that each test at which the materials are subjected requires different probes’ sizes, after the conditioning the team prepared the following samples: 30 × 30 × 6 cm for thermal conductivity test (a), 25 × 9x6cm for reaction to fire test and 16 × 4 × 6 cm for mechanical compression tests (b).

### 2.2 Density evaluation

**Fig. 6:**
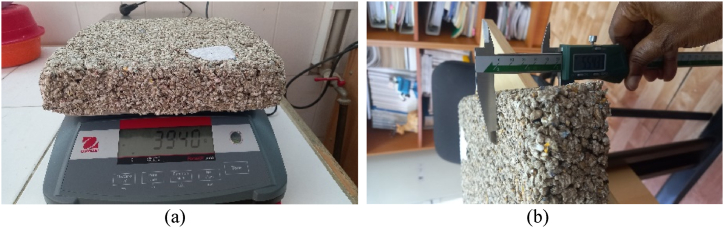
Density measurement (a) Equipment for measuring mass (b) dimensions evalation

In order to measure the material's apparent density, the volume and mass were assessed, the procedure being presented in Fig. 6. The construction materials samples were weighted by using an Ohaus RangerTM 3000 scale having a ±0,5g accuracy (a) and the dimensions-lengths, widths and thicknesses were measured with an Insize caliper code 1108-300 W with ±0.03 mm accuracy (b).

### 2.3 Thermal conductivity

**Fig. 7:**
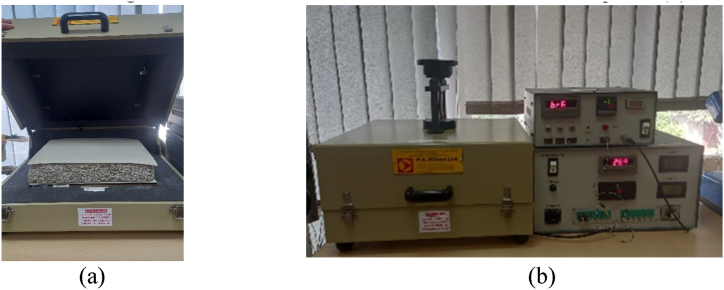
Thermal conductivity evaluation (a) Test probe placement; (b) H111 N thermal conductivity equipment.

The thermal conductivity was evaluated according to UNE EN 12667:2002 Thermal performance of building materials and products standard [21], by using a H111 N equipment produced by P.A.Hilton laid out in Fig. 7. The tests were carried out on an experimental stand authorized by the Romanian National Accreditation Body (RENAR) at the Centre of the Department of Thermal Sciences, part of the Technical University of Civil Engineering Bucharest. The EA Multilateral Agreement (EA MLA) is a signed agreement between EA members that recognizes and accepts the equivalence of the accrediting systems administered by the signing members, of which RENAR is a signatory [22]. Taking these considerations into account, all of the experimental data reported in this article can be regarded reliable.

H111 N employs a PID-controlled flat plate electric heater and a water-cooled flat plate, both of which include an integrated and very sensitive temperature sensor and also a performant heat flow meter. The sample, measuring 300 × 300 mm, is sandwiched between the heated and cooled plates- Fig. 7 (a). The maximum sample thickness is 75 mm, and the maximum temperature of the hot plate is 70 °C. To reduce heat loss, the entire system is enclosed. The supplied loading system guarantees that all specimens receive the same clamping force. The output of the heat flow meter is sent to a digital meter on the customized control and control panel (b).

### 2.4 Flexural strength

**Fig. 8:**
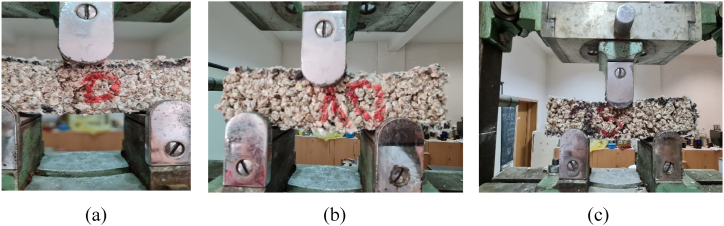
Flexural test for a clay content of (a) 0 %; (b) 10 %; (c) 25 %.

The flexural strength was determined using a three-point test on prismatic samples (Fig. 8). Flexural strength was calculated with the formula:

(1) $$f_{f}=\frac{3∙F∙l}{2∙b∙h^{2}}$$

*where: F* is the maximum load (N)

l – the span length between the supports (mm)
b-the width of the prismatic sample (mm)
h-the depth of the sample (mm).

Three prismatic samples (16 × 4 × 4 cm) were tested for each composition with a WPM universal testing machine (5000 daN). The loading direction was perpendicular to the samples' casting direction. The loading rate was about 10 N/s. The mean was calculated to the nearest 0.1 Mpa. Sample images during the three-point flexural test are presented in Fig. 8 for different percentages of clay content (a), (b), (c).

### 2.5 Reaction to fire

**Fig. 9:**
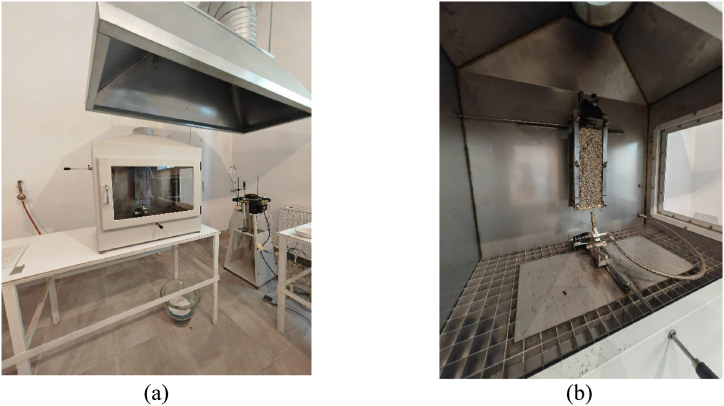
Fire testing equipment (a) General view; (b) Flame source applied on the testing probe.

Fire testing was conducted according to EN ISO 11925-2:2020 Reaction to fire tests — Ignitability [23]. The tests were also carried out on an experimental stand authorized by RENAR at INCERC Fire testing Laboratory [24] which can be observed in Fig. 9 (a). Thus, all the results can be considered trustworthy.

The procedure for testing the ignitability of items employing vertically oriented test specimens and direct small flame impingement under zero impressed irradiance are provided by standard. Fig. 9 (b) illustrates an example of test probe positioning.

The test room is capable of creating a climate of (23 ± 5) °C and (50 ± 20)% relative humidity. The combustion chamber is an enclosure made of stainless steel sheets, with access and observation points on at the front and one lateral side supplied by heat-resistant, glass doors.

The burner that serves as the ignition source is employed vertically or at a 45° angle to the vertical axis. Two flame application times are available, 30 s or 60 s. In EN ISO 11925-2:2020 paragraph 7.3.2 the standard states that the testing should last 15 s or 30 s according to the client's request.

However, we considered also a 60 s time to stress the material behavior in contact to flame. The start time of the test is established at the moment when the flame is applied.

During the test, burning particles must not be released from the material, that could lead to the ignition of a filter paper positioned in the immediate vicinity of the test sample, and also during the test, the material must not ignite on the face to which the flame is applied.

Additionally, it is important to confirm that the appropriate airflow velocity in the combustion chamber's chimney is being met; the air velocity there should be between (0.7 ± 0.1) m/s when the pilot burner is lit.

## 3 Results and discussion

### 3.1 Density

**Fig. 10:**
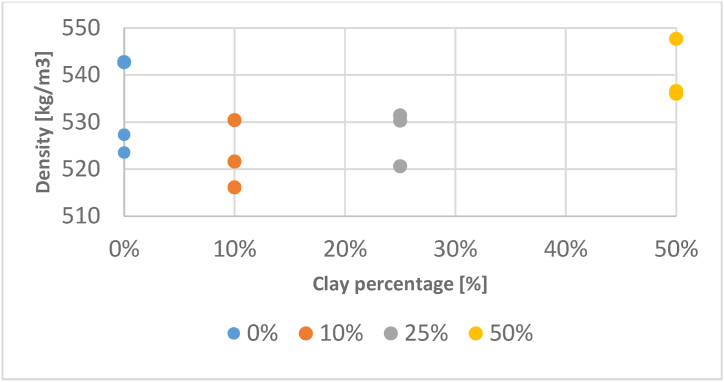
Evolution of density function of clay percentage.

The density of composites varies between extended range 515 and 550 kg/m^3^ and, in general, is a function of the percentage of clay mortar (Fig. 10).

The density of composites with 0 % clay mortar at a density between 523 and 543 kg/m^3^. When only composites containing a particular amount of clay are considered, it is clear that the higher the amount of clay mortar in the composite, the higher the density.

Thus, composites containing 10 % clay mortar are less dense, with an average density of 522.7 kg/m^3^ and a standard deviation of s = 7.21 kg/m^3^, followed by those containing 25 % clay mortar, with an average density of 527.4 kg/m^3^ with a standard deviation of s = 5.94 kg/m^3^, and composites containing 50 % clay mortar, with an average density of 540.1 kg/m^3^ and a standard deviation of s = 7.21 kg/m^3^.

### 3.2 Thermal conductivity

**Fig. 11:**
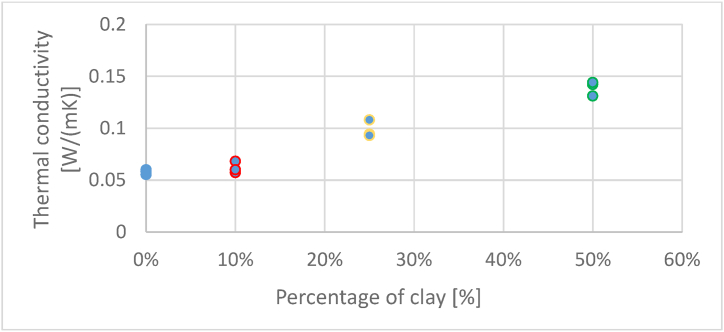
Evolution of thermal conductivity function of percentage of mortar of clay.

Fig. 11 shows that conductivity varies with clay percentage. Moving from the highest clay rate to the lowest, it is discovered that 50 % clay gives the maximum thermal conductivity, with an average value of 0.139 W/(mK) and a standard deviation s = 0.007 W/(mK). When the clay rate is reduced by half, to 25 %, the average conductivity becomes 0.0983 W/(mK) with a standard deviation s = 0.0087 W/(mK). The thermal conductivity of composites drops at an average value of 0.0627 W/(mK), and a standard deviation s = 0.0047 W/(mK) when 10 % clay is added. Finally, when considering the composition without clay, the reference composite, thermal conductivity is the lowest, falling to the average value of 0.0575 W/(mK) and a standard deviation s = 0.0023 W/(mK).

Thermal conductivity should be less than 0.1 W/(mK) for a material to be classified as an insulating material. As a result, composites containing 10 % clay and no more than 25 % clay fall into this category.

We notice that in general, conductivity increases with density. First, composites with a density between 516 and 530 kg/m^3^ (10 % clay) are the least dense and have the lowest thermal conductivity values between 0.055 … 0.06 W/(mK). Then composites with thermal conductivity values between 0.093 … 0.108 W/(mK) correspond to densities between 521 and 531 kg/m^3^. Finally, the materials with the highest densities between 540 and 550 kg/m^3^ have thermal conductivities between 0.131 … 0.144 W/(mK).

To provide a more complete view, the authors present a table comparing the thermal conductivity ratings of some commonly utilized building materials together with the values obtained in the present research [25].

**Table:** 

Material	Density [kg/m3]	Thermal conductivity [W/(m·K]
Styrofoam	10–50	0.04
Mineral wool	10–200	0.055
Wood	550	0.2
Aerated concrete block	300–1000	0.2
Cement-lime plaster	1850	0.9
Recycled paper granules + starch and 10 % clay mortar	523	0.0627
Recycled paper granules + starch and 25 % clay mortar	527	0.0983
Recycled paper granules + starch and 50 % clay mortar	540	0.139

### 3.3 Flexural strength

**Fig. 12:**
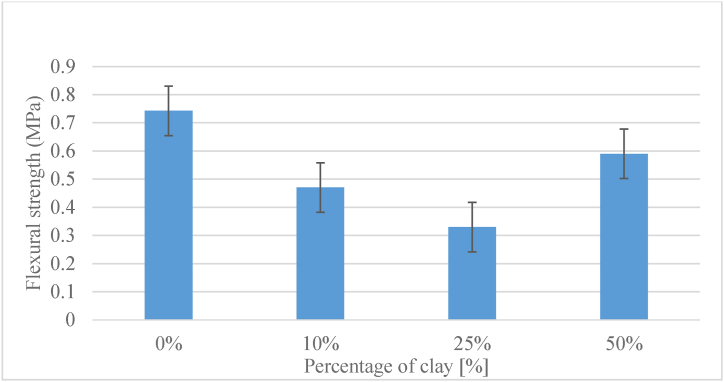
Flexural strength of tested composites.

The composites had flexural strength under 0.8 Mpa. The obtained results are presented in a graphical form in Fig. 12. The material has a flexural strength of 0.74 with 0 % clay content, showing that the pure base material has strong inherent strength. The decrease in flexural strength as clay concentration increases is notable, with a significant dip to 0.47 at 10 % clay content. This initial decrease could be related to the addition of clay particles, which may be less resilient than the base material.

As the clay percentage grows to 25 %, the flexural strength continues to fall to 0.33. This trend suggests that the clay, while possibly enhancing certain properties such as plasticity, may be diminishing the material's ability to withstand bending forces, possibly due to its brittle.

Interestingly, at 50 % clay content, there is a slight increase in flexural strength to 0.59 compared to the 25 % clay content. This deviation may be due to the complex interplay of various factors, including clay particle size, dispersion, and the bonding between clay and the base material.

### 3.4 Reaction to fire

**Fig. 13:**
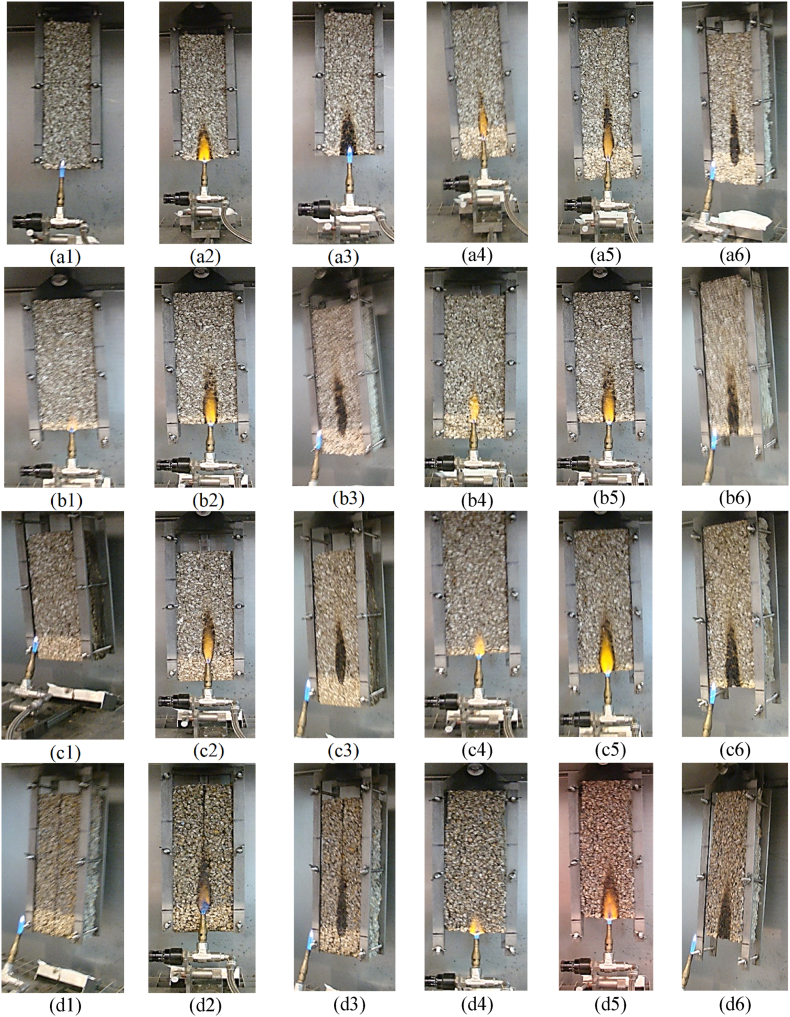
Materials' exposure to standard flame (a) 0 %; (b) 10 %; (c) 25 %; (d) 50 %.

All tested samples ignited upon contact with the standard flame, and after its removal, the flame did not self-maintain and extinguish in any of the four studied compositions both on the edge and on the materials’ surface as it can be spotted in Fig. 13. The flame did not propagate to a height greater than 15 cm during the 60 s test time, so it can be concluded that the materials match minimally in the class E of reaction to fire.

During the tests, no burning particles were released from the material that would lead to the ignition of the filter paper, so the materials are considered to have passed the ignitability test.

In general, without noticeable differences, a similar behavior to the action of the standard flame was observed for all four types of materials in terms of the moment of flame initiation, the height of propagation and its extinction after removing the standard flame.

### 3.5 1-3 edge testing; 4–6 surface testing

Fig. 13(a–d) presents for each clay percentage 0 %, 10 %, 25 % and 50 % respectively diferent steps during fire exposure test. Thus, the first three pictures for each percentage refers to the edge testing, initial phase (a1), (b1), (c1), (d1) an intermediate stage (a2), (b2), (c2), (d2) and final phase (a3), (b3), (c3) and (d3). Correspondingly, the following three pictures from each row display the surface testing, initial phase (a4), (b4), (c4), (d4), an intermediate stage (a5), (b5), (c5), (d5) and the final phase (a6), (b6), (c6) and (d6).

**Fig. 14:**
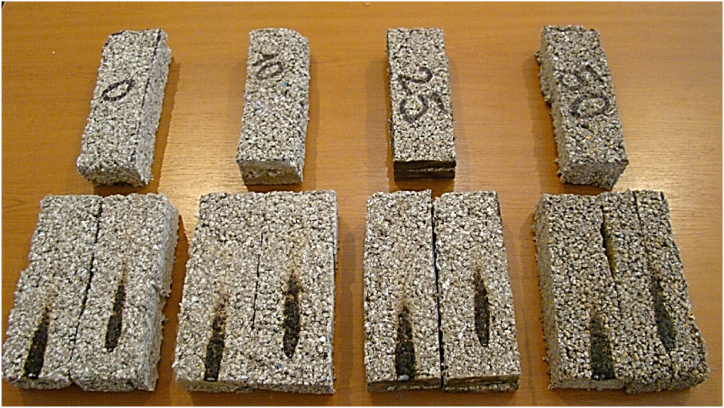
Materials' appearance following testing.

Fig. 14 depicts the materials’ appearance following testing.

The product tested for reaction to fire was classified in minimum reaction to fire class E, in accordance with the provisions of the Standard for fire classification of products and construction elements EN 13501-1:2019. During the testing of the samples to the action of the standard flame according to EN ISO 11925-2:2020, the smoke emission is not measured and for the classification of the tested product in the reaction to fire class E, the classification standard does not provide a classification either in terms of smoke released during the tests. According to this standard, only the height of the flame and the appearance of burning droplets or particles that come off the test probes are evaluated. For the material tested in fire, no burning droplets or particles were observed.

## 4 Conclusions

This work deals with the development of new types of materials composed of paper waste, starch and by varying the proportion of clay mortar from 0 to 50 %. The main advantage of mixing paper waste and starch is given by the fact that they are often cost-effective materials, potentially reducing production costs compared to traditional materials. This can make the new composites economically attractive, particularly in poor regions where these raw materials are readily available. Utilizing locally available paper waste and starch can reduce the carbon footprint associated with transportation and the extraction of raw materials, contributing to a more sustainable and eco-friendly material production process. The development of such materials fosters innovation and is consistent with the concepts of a circular economy, in which waste materials are upcycled into valuable products, minimizing the demand for virgin resources.

A disadvantage could be the fact that materials with higher paper waste and starch content have limited load-bearing capacity and may not be suitable for applications requiring substantial strength, such as load-bearing construction elements.

Considering the fact that the presented material represents an inovation, at the moment there aren't direct standards for testing and evaluation. Thus, in order to form an opinion, the flexural-breaking load of the mortars was compared to the gypsum plasterboard, a relatively common material in the construction sector. According to DIN 18180:2014, the flexural breaking load of the gypsum plasterboard (thickness of 12.5 mm) in the transversal direction has to be ≥ 210 N. The prepared mortars had flexural breaking load in the range of 260 … 480 N. The material's primary application is in interior cladding boards or complex panel assemblies designed to achieve high energy efficiency.

All analyzed material compositions passed the 60s ignitability test and behaved similarly, which highlights the fact that the percentage of clay in the composition does not have a particular influence on fire behavior. More than that, a conclusion that can be drawn from the test is that the material falls, regardless of composition, at least in fire reaction class E. In order to establish the fire reaction class as accurately as possible in the future in the research that will follow the authors intend to carry out more complex tests on the behavior of the material in fire including the determination of the heat of combustion in a bomb calorimeter.

The determination of the thermal conductivity of composite materials clearly shows an upward trend of its value with the increase in percentage of clay. Following the research conducted, it can be recommended that the material with a percentage of 10 % clay can be used in buildings wall structures due to the good insulating properties, having an average thermal conductivity of 0.0627 W/(mK), and a standard deviation s = 0.0047 W/(mK). When compared to the results of other studies, the thermal conductivity value is consistent with materials considered to have an insulating role [19,25].

Regarding the flexural strength, it can be concluded that the material with 10 % clay gives the best results, after that it becomes brittle. To better understand the phenomenon of the increasing value corresponding to 50 % clay in mixture, the authors take into consideration to investigate in future research the microstructural changes and interactions that occur inside the composite as the clay content varies and also to conduct a porosity analysis that could give some insight into the mechanisms underlying the flexural strength changes.

The research presented is in line with global trends toward environmental preservation, recycling materials used in construction and other fields of activity, and achieving thermal comfort and security for inhabitants of residences. The authors plan to examine the hygric properties due to hydrophilic nature of material and other composite material structures in the future that have little or no adverse environmental effects.

## Additional information

No additional information is available for this paper.

## CRediT authorship contribution statement

**Lydie Marcelle Thieblesson:** Writing – review & editing, Writing – original draft, Methodology, Investigation, Data curation, Conceptualization. **Răzvan Calotă:** Writing – review & editing, Writing – original draft, Project administration, Methodology, Investigation, Formal analysis, Data curation, Conceptualization. **Nastasia Saca:** Writing – review & editing, Writing – original draft, Methodology, Investigation. **Adrian Simion:** Writing – original draft, Validation, Resources, Conceptualization. **Ilinca Năstase:** Writing – original draft, Supervision, Resources, Project administration, Conceptualization. **Alina Girip:** Writing – review & editing, Writing – original draft, Investigation.

## Declaration of competing interest

The authors declare the following financial interests/personal relationships which may be considered as potential competing interests:Lydie THIEBLESSON reports financial support was provided by 10.13039/501100002708Agence universitaire de la Francophonie.
